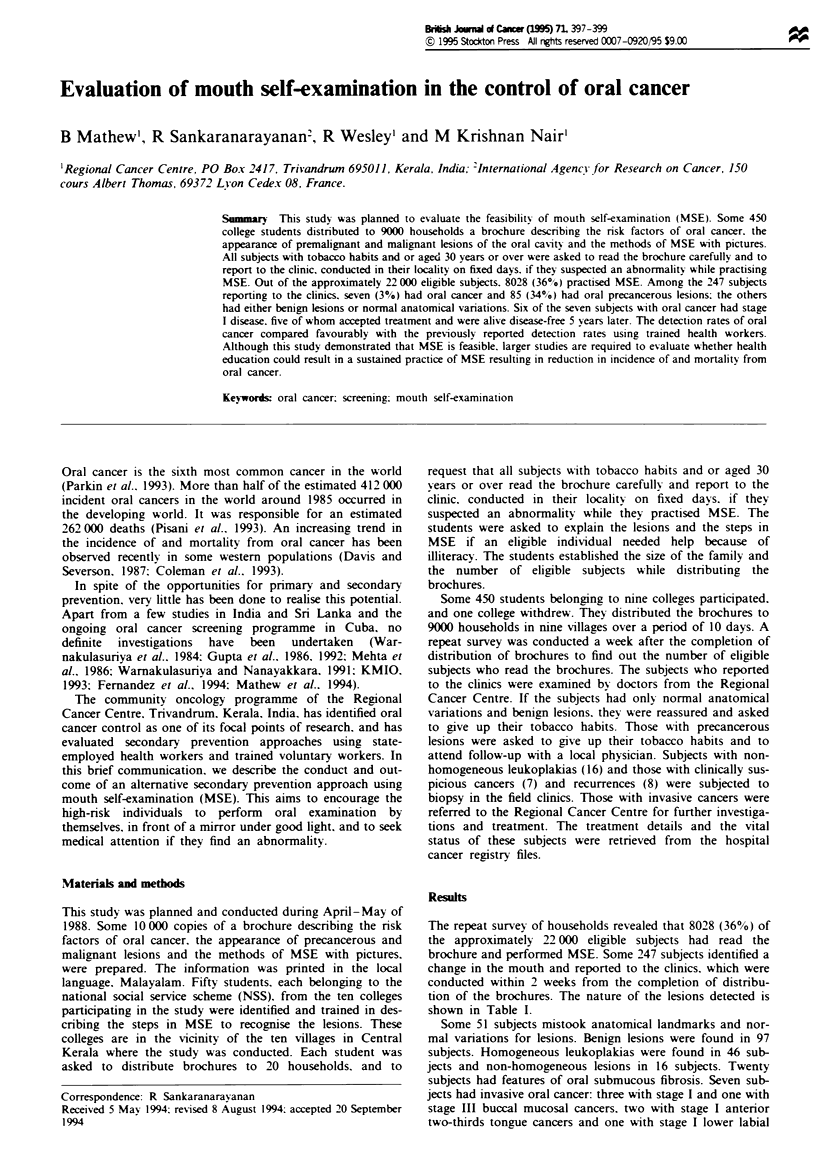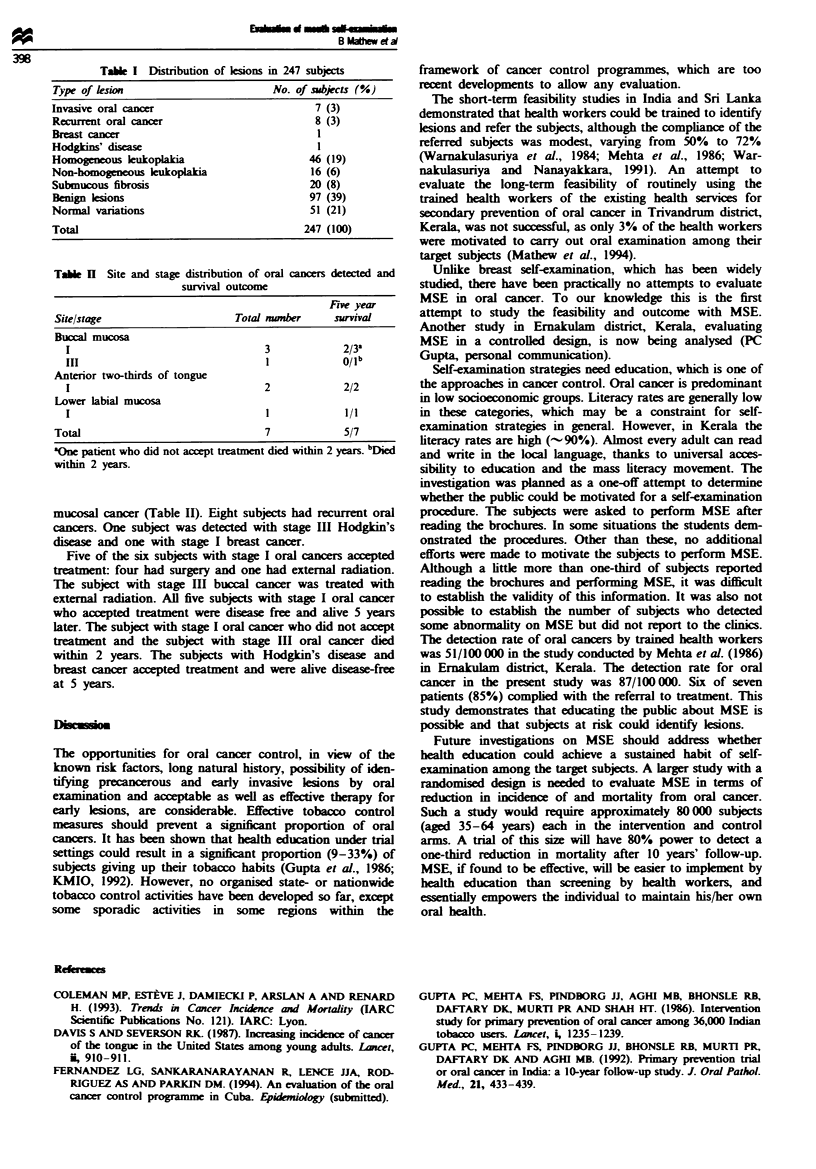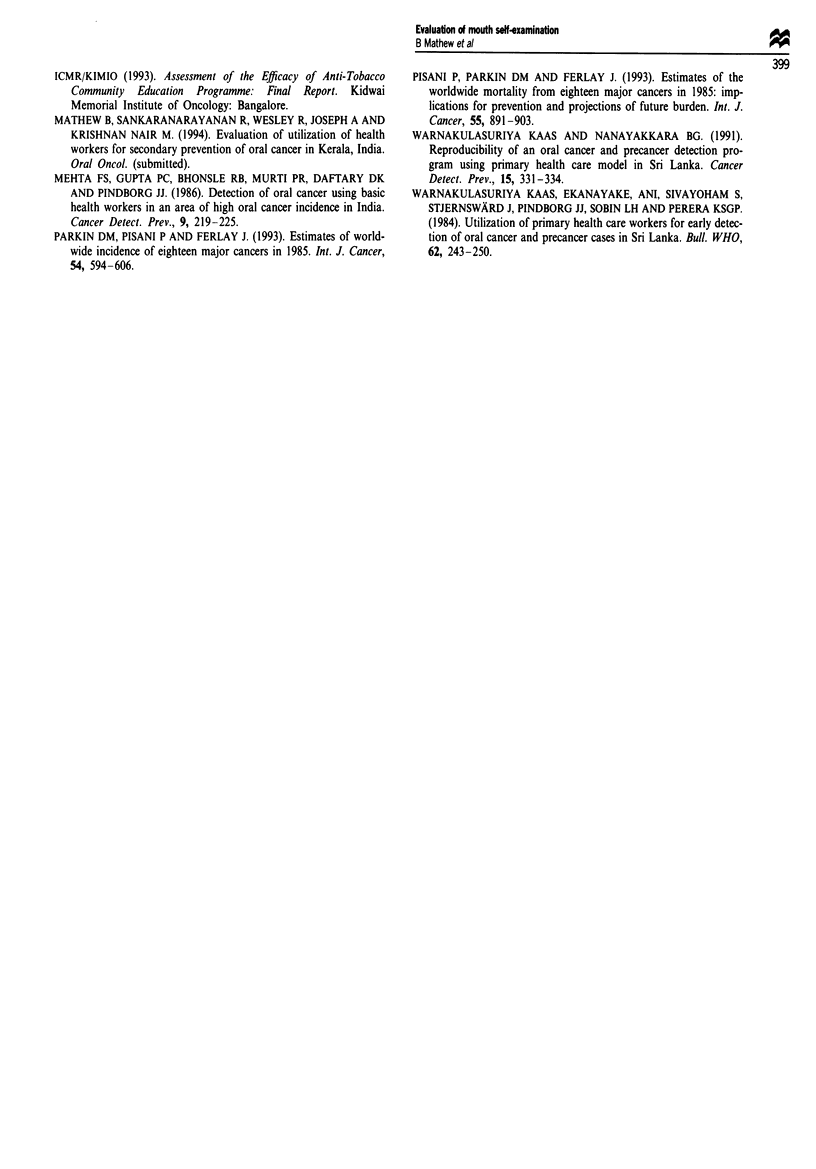# Evaluation of mouth self-examination in the control of oral cancer.

**DOI:** 10.1038/bjc.1995.81

**Published:** 1995-02

**Authors:** B. Mathew, R. Sankaranarayanan, R. Wesley, M. K. Nair

**Affiliations:** Regional Cancer Centre, Kerala, India.

## Abstract

This study was planned to evaluate the feasibility of mouth self-examination (MSE). Some 450 college students distributed to 9000 households a brochure describing the risk factors of oral cancer, the appearance of premalignant and malignant lesions of the oral cavity and the methods of MSE with pictures. All subjects with tobacco habits and/or aged 30 years or over were asked to read the brochure carefully and to report to the clinic conducted in their locality on fixed days, if they suspected an abnormality while practising MSE. Out of the approximately 22,000 eligible subjects, 8028 (36%) practised MSE. Among the 247 subjects reporting to the clinics seven (3%) had oral cancer and 85 (34%) had oral precancerous lesions; the others had either benign lesions or normal anatomical variations. Six of the seven subjects with oral cancer had stage I disease, five of whom accepted treatment and were alive disease-free 5 years later. The detection rates of oral cancer compared favourably with the previously reported detection rates using trained health workers. Although this study demonstrated that MSE is feasible, larger studies are required to evaluate whether health education could result in a sustained practice of MSE resulting in reduction in incidence of and mortality from oral cancer.


British Journal of Cancer (1995) 71, 397-399

© 1995 Stockton Press All rights reserved 0007-0920/95 $9.00

### Evaluation of mouth self-examination in the control of oral cancer

B Mathew[1], R Sankaranarayanan[2], R Wesley[1] and M Krishnan Nair[1]

[1]*Regional Cancer Centre, PO Box 2417, Trivandrum 695011, Kerala, India;* [2]*International Agency for Research on Cancer, 150 cours Albert Thomas, 69372 Lyon Cedex 08, France.*

**Summary** This study was planned to evaluate the feasibility of mouth self-examination (MSE). Some 450 college students distributed to 9000 households a brochure describing the risk factors of oral cancer, the appearance of premalignant and malignant lesions of the oral cavity and the methods of MSE with pictures. All subjects with tobacco habits and/or aged 30 years or over were asked to read the brochure carefully and to report to the clinic, conducted in their locality on fixed days, if they suspected an abnormality while practising MSE. Out of the approximately 22 000 eligible subjects, 8028 (36%) practised MSE. Among the 247 subjects reporting to the clinics, seven (3%) had oral cancer and 85 (34%) had oral precancerous lesions; the others had either benign lesions or normal anatomical variations. Six of the seven subjects with oral cancer had stage I disease, five of whom accepted treatment and were alive disease-free 5 years later. The detection rates of oral cancer compared favourably with the previously reported detection rates using trained health workers. Although this study demonstrated that MSE is feasible, larger studies are required to evaluate whether health education could result in a sustained practice of MSE resulting in reduction in incidence of and mortality from oral cancer.

**Keywords:** oral cancer; screening; mouth self-examination

Oral cancer is the sixth most common cancer in the world (Parkin *et al.*, 1993). More than half of the estimated 412 000 incident oral cancers in the world around 1985 occurred in the developing world. It was responsible for an estimated 262 000 deaths (Pisani *et al.*, 1993). An increasing trend in the incidence of and mortality from oral cancer has been observed recently in some western populations (Davis and Severson, 1987; Coleman *et al.*, 1993).

In spite of the opportunities for primary and secondary prevention, very little has been done to realise this potential. Apart from a few studies in India and Sri Lanka and the ongoing oral cancer screening programme in Cuba, no definite investigations have been undertaken (Warnakulasuriya *et al.*, 1984; Gupta *et al.*, 1986, 1992; Mehta *et al.*, 1986; Warnakulasuriya and Nanayakkara, 1991; KMIO, 1993; Fernandez *et al.*, 1994; Mathew *et al.*, 1994).

The community oncology programme of the Regional Cancer Centre, Trivandrum, Kerala, India, has identified oral cancer control as one of its focal points of research, and has evaluated secondary prevention approaches using state-employed health workers and trained voluntary workers. In this brief communication, we describe the conduct and outcome of an alternative secondary prevention approach using mouth self-examination (MSE). This aims to encourage the high-risk individuals to perform oral examination by themselves, in front of a mirror under good light, and to seek medical attention if they find an abnormality.

### Materials and methods

This study was planned and conducted during April–May of 1988. Some 10 000 copies of a brochure describing the risk factors of oral cancer, the appearance of precancerous and malignant lesions and the methods of MSE with pictures, were prepared. The information was printed in the local language, Malayalam. Fifty students, each belonging to the national social service scheme (NSS), from the ten colleges participating in the study were identified and trained in describing the steps in MSE to recognise the lesions. These colleges are in the vicinity of the ten villages in Central Kerala where the study was conducted. Each student was asked to distribute brochures to 20 households, and to request that all subjects with tobacco habits and/or aged 30 years or over read the brochure carefully and report to the clinic, conducted in their locality on fixed days, if they suspected an abnormality while they practised MSE. The students were asked to explain the lesions and the steps in MSE if an eligible individual needed help because of illiteracy. The students established the size of the family and the number of eligible subjects while distributing the brochures.

Some 450 students belonging to nine colleges participated, and one college withdrew. They distributed the brochures to 9000 households in nine villages over a period of 10 days. A repeat survey was conducted a week after the completion of distribution of brochures to find out the number of eligible subjects who read the brochures. The subjects who reported to the clinics were examined by doctors from the Regional Cancer Centre. If the subjects had only normal anatomical variations and benign lesions, they were reassured and asked to give up their tobacco habits. Those with precancerous lesions were asked to give up their tobacco habits and to attend follow-up with a local physician. Subjects with non-homogeneous leukoplakias (16) and those with clinically suspicious cancers (7) and recurrences (8) were subjected to biopsy in the field clinics. Those with invasive cancers were referred to the Regional Cancer Centre for further investigations and treatment. The treatment details and the vital status of these subjects were retrieved from the hospital cancer registry files.

### Results

The repeat survey of households revealed that 8028 (36%) of the approximately 22 000 eligible subjects had read the brochure and performed MSE. Some 247 subjects identified a change in the mouth and reported to the clinics, which were conducted within 2 weeks from the completion of distribution of the brochures. The nature of the lesions detected is shown in Table I.

Some 51 subjects mistook anatomical landmarks and normal variations for lesions. Benign lesions were found in 97 subjects. Homogeneous leukoplakias were found in 46 subjects and non-homogeneous lesions in 16 subjects. Twenty subjects had features of oral submucous fibrosis. Seven subjects had invasive oral cancer: three with stage I and one with stage III buccal mucosal cancers, two with stage I anterior two-thirds tongue cancers and one with stage I lower labial

Correspondence: R Sankaranarayanan
Received 5 May 1994; revised 8 August 1994; accepted 20 September 1994

**Table I** Distribution of lesions in 247 subjects

| *Type of lesion* | *No. of subjects (%)* |
|---|---|
| Invasive oral cancer | 7 (3) |
| Recurrent oral cancer | 8 (3) |
| Breast cancer | 1 |
| Hodgkins' disease | 1 |
| Homogeneous leukoplakia | 46 (19) |
| Non-homogeneous leukoplakia | 16 (6) |
| Submucous fibrosis | 20 (8) |
| Benign lesions | 97 (39) |
| Normal variations | 51 (21) |
| Total | 247 (100) |

**Table II** Site and stage distribution of oral cancers detected and survival outcome

| *Site stage* | *Total number* | *Five year survival* |
|---|---|---|
| Buccal mucosa | | |
| I | 3 | 2 3[a] |
| III | 1 | 0 1[b] |
| Anterior two-thirds of tongue | | |
| I | 2 | 2 2 |
| Lower labial mucosa | | |
| I | 1 | 1 1 |
| Total | 7 | 5 7 |

[a]One patient who did not accept treatment died within 2 years. [b]Died within 2 years.

mucosal cancer (Table II). Eight subjects had recurrent oral cancers. One subject was detected with stage III Hodgkin's disease and one with stage I breast cancer.

Five of the six subjects with stage I oral cancers accepted treatment: four had surgery and one had external radiation. The subject with stage III buccal cancer was treated with external radiation. All five subjects with stage I oral cancer who accepted treatment were disease free and alive 5 years later. The subject with stage I oral cancer who did not accept treatment and the subject with stage III oral cancer died within 2 years. The subjects with Hodgkin's disease and breast cancer accepted treatment and were alive disease-free at 5 years.

### Discussion

The opportunities for oral cancer control, in view of the known risk factors, long natural history, possibility of identifying precancerous and early invasive lesions by oral examination and acceptable as well as effective therapy for early lesions, are considerable. Effective tobacco control measures should prevent a significant proportion of oral cancers. It has been shown that health education under trial settings could result in a significant proportion (9–33%) of subjects giving up their tobacco habits (Gupta *et al*., 1986; KMIO, 1992). However, no organised state- or nationwide tobacco control activities have been developed so far, except some sporadic activities in some regions within the framework of cancer control programmes, which are too recent developments to allow any evaluation.

The short-term feasibility studies in India and Sri Lanka demonstrated that health workers could be trained to identify lesions and refer the subjects, although the compliance of the referred subjects was modest, varying from 50% to 72% (Warnakulasuriya *et al*., 1984; Mehta *et al*., 1986; Warnakulasuriya and Nanayakkara, 1991). An attempt to evaluate the long-term feasibility of routinely using the trained health workers of the existing health services for secondary prevention of oral cancer in Trivandrum district, Kerala, was not successful, as only 3% of the health workers were motivated to carry out oral examination among their target subjects (Mathew *et al*., 1994).

Unlike breast self-examination, which has been widely studied, there have been practically no attempts to evaluate MSE in oral cancer. To our knowledge this is the first attempt to study the feasibility and outcome with MSE. Another study in Ernakulam district, Kerala, evaluating MSE in a controlled design, is now being analysed (PC Gupta, personal communication).

Self-examination strategies need education, which is one of the approaches in cancer control. Oral cancer is predominant in low socioeconomic groups. Literacy rates are generally low in these categories, which may be a constraint for self-examination strategies in general. However, in Kerala the literacy rates are high (~90%). Almost every adult can read and write in the local language, thanks to universal accessibility to education and the mass literacy movement. The investigation was planned as a one-off attempt to determine whether the public could be motivated for a self-examination procedure. The subjects were asked to perform MSE after reading the brochures. In some situations the students demonstrated the procedures. Other than these, no additional efforts were made to motivate the subjects to perform MSE. Although a little more than one-third of subjects reported reading the brochures and performing MSE, it was difficult to establish the validity of this information. It was also not possible to establish the number of subjects who detected some abnormality on MSE but did not report to the clinics. The detection rate of oral cancers by trained health workers was 51 100 000 in the study conducted by Mehta *et al*. (1986) in Ernakulam district, Kerala. The detection rate for oral cancer in the present study was 87 100 000. Six of seven patients (85%) complied with the referral to treatment. This study demonstrates that educating the public about MSE is possible and that subjects at risk could identify lesions.

Future investigations on MSE should address whether health education could achieve a sustained habit of self-examination among the target subjects. A larger study with a randomised design is needed to evaluate MSE in terms of reduction in incidence of and mortality from oral cancer. Such a study would require approximately 80 000 subjects (aged 35–64 years) each in the intervention and control arms. A trial of this size will have 80% power to detect a one-third reduction in mortality after 10 years' follow-up. MSE, if found to be effective, will be easier to implement by health education than screening by health workers, and essentially empowers the individual to maintain his her own oral health.

### References

COLEMAN MP, ESTÈVE J, DAMIECKI P, ARSLAN A AND RENARD H. (1993). *Trends in Cancer Incidence and Mortality* (IARC Scientific Publications No. 121). IARC: Lyon.

DAVIS S AND SEVERSON RK. (1987). Increasing incidence of cancer of the tongue in the United States among young adults. *Lancet*, **ii**, 910–911.

FERNANDEZ LG, SANKARANARAYANAN R, LENCE JJA, RODRIGUEZ AS AND PARKIN DM. (1994). An evaluation of the oral cancer control programme in Cuba. *Epidemiology* (submitted).

GUPTA PC, MEHTA FS, PINDBORG JJ, AGHI MB, BHONSLE RB, DAFTARY DK, MURTI PR AND SHAH HT. (1986). Intervention study for primary prevention of oral cancer among 36,000 Indian tobacco users. *Lancet*, **i**, 1235–1239.

GUPTA PC, MEHTA FS, PINDBORG JJ, BHONSLE RB, MURTI PR, DAFTARY DK AND AGHI MB. (1992). Primary prevention trial or oral cancer in India: a 10-year follow-up study. *J. Oral Pathol. Med.*, **21**, 433–439.

ICMR/KIMIO (1993). *Assessment of the Efficacy of Anti-Tobacco Community Education Programme: Final Report.* Kidwai Memorial Institute of Oncology: Bangalore.

MATHEW B, SANKARANARAYANAN R, WESLEY R, JOSEPH A AND KRISHNAN NAIR M. (1994). Evaluation of utilization of health workers for secondary prevention of oral cancer in Kerala, India. *Oral Oncol.* (submitted).

MEHTA FS, GUPTA PC, BHONSLE RB, MURTI PR, DAFTARY DK AND PINDBORG JJ. (1986). Detection of oral cancer using basic health workers in an area of high oral cancer incidence in India. *Cancer Detect. Prev.*, **9**, 219-225.

PARKIN DM, PISANI P AND FERLAY J. (1993). Estimates of worldwide incidence of eighteen major cancers in 1985. *Int. J. Cancer*, **54**, 594-606.

PISANI P, PARKIN DM AND FERLAY J. (1993). Estimates of the worldwide mortality from eighteen major cancers in 1985: implications for prevention and projections of future burden. *Int. J. Cancer*, **55**, 891-903.

WARNAKULASURIYA KAAS AND NANAYAKKARA BG. (1991). Reproducibility of an oral cancer and precancer detection program using primary health care model in Sri Lanka. *Cancer Detect. Prev.*, **15**, 331-334.

WARNAKULASURIYA KAAS, EKANAYAKE, ANI, SIVAYOHAM S, STJERNSWÄRD J, PINDBORG JJ, SOBIN LH AND PERERA KSGP. (1984). Utilization of primary health care workers for early detection of oral cancer and precancer cases in Sri Lanka. *Bull. WHO*, **62**, 243-250.